# Efficacy of Low-Dose Streptokinase Infusion in Late-Onset Permanent Tunnel Catheter Dysfunction: A Single-Center Interventional Study

**DOI:** 10.7759/cureus.58028

**Published:** 2024-04-11

**Authors:** Kshitija Gadekar, Saif Zil Kibriya, Pranav Kulkarni, Sowntappan Balasubramanian

**Affiliations:** 1 Department of Nephrology, Mahatma Gandhi Mission (MGM) Medical College and Hospital, Aurangabad, IND; 2 Department of Community Medicine, Seth Gordhandas Sunderdas Medical College and King Edward Memorial Hospital, Mumbai, IND

**Keywords:** hemodialysis, thrombolytic therapy, resource limited settings, catheters, streptokinase

## Abstract

Introduction

Hemodialysis is a vital modality for patients with renal dysfunction, with venous access being a significant factor in its success. While arteriovenous fistulas are preferred, tunneled catheters serve as important alternatives, especially in challenging cases. Late-onset tunneled catheter dysfunction, often due to fibrin sheath formation, impedes hemodialysis efficiency. Streptokinase, a low-cost thrombolytic agent, has shown promise in resolving such complications, yet its efficacy in the Indian context remains unexplored.

Methods

We conducted a single-center interventional study at Mahatma Gandhi Mission (MGM) Hospital, Aurangabad, India, from May 2023 to October 2023. Ethical approval was obtained, and 10 eligible patients experiencing late-onset permanent tunnel catheter dysfunction were enrolled. Patients were treated with low-dose streptokinase, and outcomes were monitored for 60 days.

Results

Ten patients, evenly distributed by gender, participated, with a mean age of 48.2 ± 11.96 years. Diabetes was the predominant cause of chronic kidney disease (CKD) at 33% (3/10). All patients achieved the primary endpoint of blood flow rate (BFR) >300 ml/min post-streptokinase treatment, with an overall success rate of 100%. Group A had the highest average gain in catheter days (80.6 ± 7.59), followed by Group B (64 ± 1), while Group C showed variations in catheter days between the first (26.2 ± 6.8) and second insertion (32.5 ± 1.76). Eight patients maintained catheter patency during the 60-day follow-up. Adverse effects, primarily minor, were observed. The dosage rationale involved an eight-hour infusion at 4,000 units per hour.

Conclusion

Streptokinase emerges as cost-effective and efficacious for maintaining the patency of late-onset tunnel catheter dysfunction in resource-limited settings, particularly in younger patients. Caution is advised for older individuals with prolonged CKD.

## Introduction

Venous access is the Achilles’ heel of hemodialysis, which remains the modality of paramount importance [1]. As per the Kidney Disease Outcomes Quality Initiative (KDOQI) 2019 vascular access guidelines, the arteriovenous (AV) fistula remains the venous access of choice in view of its low complication rates [2]. However, AV fistulas require six weeks for maturation and may not be possible in some patients with sclerotic vessels and long-term diabetes [3]. Tunneled catheters serve both as a bridge to the AV fistula and as primary and only access in some patients. However, catheter dysfunction in hemodialysis patients reduces blood flow, leading to lower dialysis efficiency [4]. Permanent catheter dysfunction can be early, arising in the first few weeks (less than four weeks), or late. Early dysfunction is commonly due to the malposition of the catheter tip and subsequent thrombosis, while late-onset tunneled catheter dysfunction is commonly due to fibrin sheath formation [5]. The KDOQI 2019 guidelines support the use of intraluminal thrombolysis in the setting of thrombosed catheters, as fibrin sheath formation can exacerbate catheter dysfunction [6]. In resource-limited settings, alteplase, the FDA-approved drug, can be costly, whereas streptokinase in a low-dose infusion can be used for such catheters. Reports of the use of streptokinase for blocked permanent tunneled catheters have been found to be effective and have been reported from Asia and around the world [7,8]. This study aims to evaluate the efficacy of low-dose streptokinase infusion in late-onset permanent tunnel catheter dysfunction. We report the first such study evaluating the use of a low-dose streptokinase infusion for blocked tunneled permanent catheters from South India.

## Materials and methods

We conducted the interventional study at Mahatma Gandhi Mission (MGM) Hospital in Aurangabad, India, from May 2023 to October 2023. We selected 10 patients with late-onset perm catheter dysfunction for the study based on the inclusion criteria. The study commenced after approval from the Ethical Review Board Committee of the hospital.

Inclusion criteria

All patients must be over the age of 18 and willing to take streptokinase as per protocol. Patients with late-onset tunneled permanent catheter dysfunction, which is defined as failure to attain and maintain an extracorporeal blood flow, Qb, of 300 ml/min or greater at a pre-pump arterial pressure more negative than -250 mm Hg.

Exclusion criteria

Patients unwilling to follow the low-dose streptokinase protocol who have other options for venous access. Patients with contraindications to streptokinase use, including severe uncontrolled hypertension, intracranial neoplasms, surgery within two months, recent stroke, intraspinal surgery, active internal bleeding, pregnancy, evidence, or history of coagulopathy. Patients taking maintenance hemodialysis outside our center.

Study procedure

The study commenced after obtaining well-informed and written consent from the patients. Patients were given a low-dose streptokinase infusion at 4,000 units per hour in both ports for eight hours (MGM protocol). We noted the side effects during and after the procedure. The primary endpoint was the establishment of a blood flow rate (BFR) >300 ml after a streptokinase protocol dose in a previously blocked tunneled perm catheter. Following the primary endpoint, the secondary endpoint involves a 60-day follow-up period wherein the patency of a previously blocked permanent catheter is checked at the 30th, 45th, and 60th days.

We divided the patients into three groups (A, B, and C) based on their clinical profile and repeated use of streptokinase. Group A consists of patients whose tunneled catheter was blocked for the first time and required only one administration of the protocol dose. Group B consists of patients whose tunneled catheters were blocked, who previously received urokinase, and who required only one protocol dose administration. Group C comprises patients whose tunneled catheter was blocked previously and who used urokinase before but required repeated administration of the protocol dose.

We observed adverse effects during and after the streptokinase protocol installation, dividing them into major and minor categories. Major effects include cerebrovascular accidents, hemopericardium, hemoperitoneum, anaphylaxis, severe hypotension requiring IV fluid or stoppage of infusion, and arrhythmias. Minor adverse effects include fever with chills, hematuria, bleeding from orifices or catheter sites, tremors, bradycardia, and headache.

Patency was defined as blood flow of about 300 ml/min, which persisted for about 30 minutes during the hemodialysis session after thrombolysis.

Statistical analysis

Statistical analysis was performed using IBM SPSS Statistics for Windows, Version 24.0 (Released 2016; IBM Corp., Armonk, NY, USA). The qualitative data were described in frequency and percentage. The quantitative data were described as mean and standard deviation.

## Results

Demographics details

A total of 10 patients participated in the study, with an equal distribution of five males and five females. The mean age of all the patients was 48.2 ± 11.96 years. The mean age of patients in Group A, Group B, and Group C was 39 ± 3.08, 58 ± 8, and 66 ± 2 years, respectively. The average duration of chronic kidney disease (CKD) was 16.4 ± 13 months. Diabetes mellitus, accounting for 33% (3/10), was the most common cause of CKD, followed by obstructive uropathy at 20% (2/10) and autosomal dominant polycystic kidney disease at 20% (2/10).

Catheter patency

**Figure 1:**
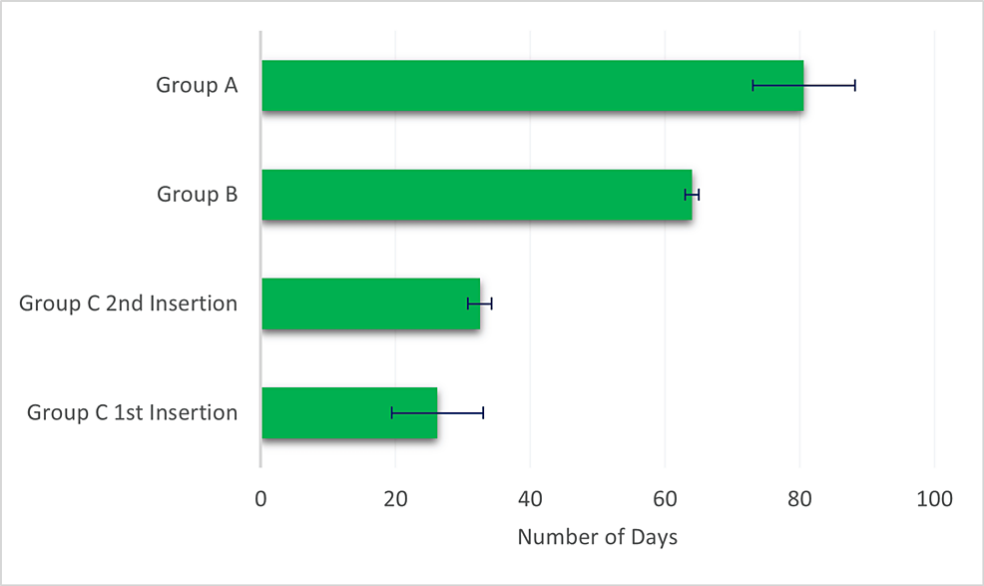
Comparison of the average gain in catheter days among different groups

Prior to catheter blockade, the average duration of catheter half-life was 138.6 ± 84.69 days. Following the administration of streptokinase, Group A exhibited an average gain of catheter days of 80.6 ± 7.59, Group B had 64 ± 1, and Group C exhibited variations between the first insertion (26.2 ± 6.8) and the second insertion (32.5 ± 1.76), as mentioned in Figure 1.

All patients achieved the primary endpoint, with a BFR >300 ml/min, resulting in a 100% success rate (10/10). Additionally, 80% of patients (8/10) achieved the secondary endpoint, maintaining catheter patency during the 60-day follow-up.

Adverse effects

**Figure 2:**
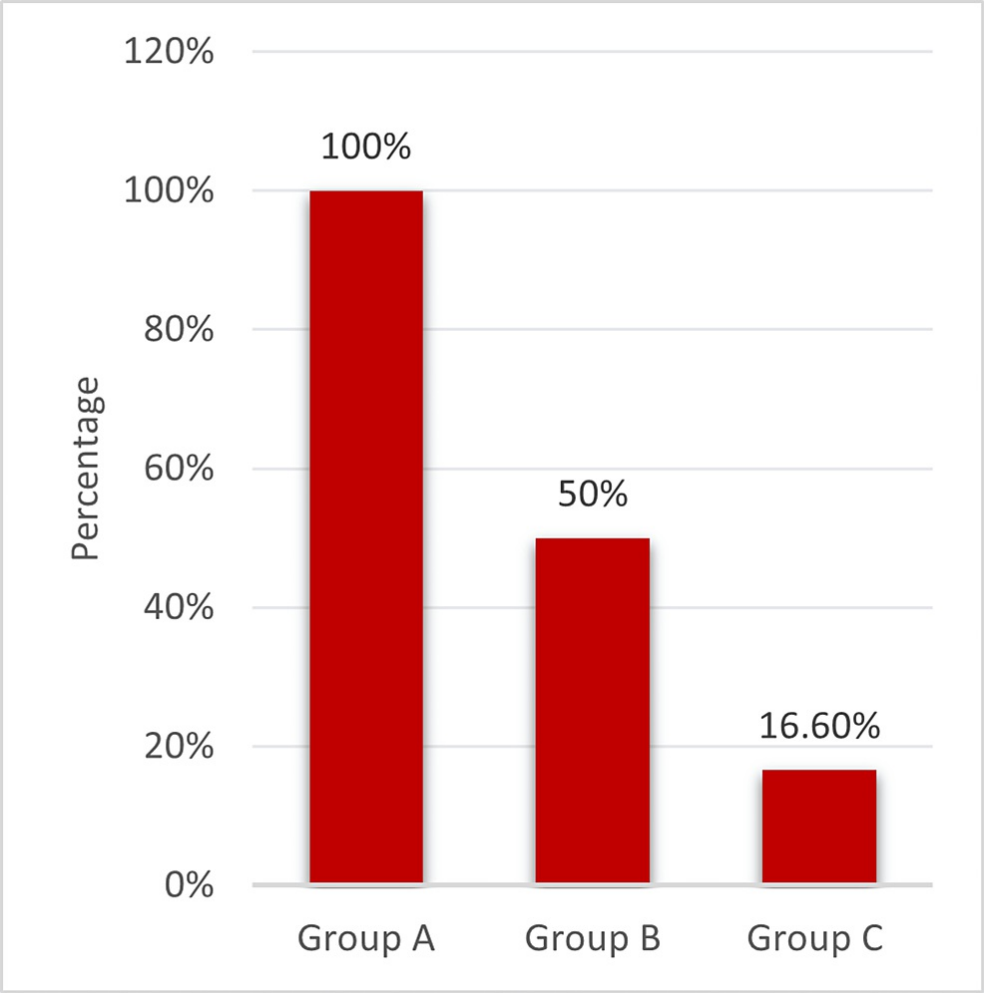
Incidence of adverse events

We observed the adverse effects as mentioned in Figure 2, and it was found that Group C 100% (2/2) showed a higher incidence when compared to Group A 16.66% (1/6) and Group B 50% (1/2). This finding could be explained by the older age of Group C (mean age: 66 ± 2 years) participants compared with Group A (mean age: 39 ± 3 years).

## Discussion

Tunneled catheters serve as a bridge before the maturation of the AV fistula, and in many patients with multiple access failures, they may be the only access for hemodialysis; hence, their blockage due to thrombosis or fibrin formation can be a catastrophic event. Streptokinase, the polypeptide derived from beta-hemolytic streptococci, forms a complex with plasminogen, initiating a cascade that then converts into the proteolytic enzyme plasmin [9]. This process facilitates the lysis of fibrin clots, providing the rationale for its use in addressing late-onset permanent catheter (perm catheter) dysfunction associated with fibrin sheath formation [10]. The pioneering work of Welik et al. in 1987, utilizing repeated low-dose streptokinase for occluded catheters, laid the basis for exploring this therapeutic opportunity [11].

While alteplase, reteplase, and urokinase have well-established roles in restoring the patency of occluded HD catheters [10], there have been limited studies on the use and dosing regimen of streptokinase, with the exception of a few case-controlled studies from Asia and a recent study from India [7,8]. Our study is the first interventional investigation of streptokinase use as a low-dose infusion for occluded tunneled permanent catheters from the western part of India, highlighting its potential economic advantages.

Streptokinase (1.5 MI units) proves to be a cost-effective alternative, with a vial costing 900 INR, significantly more affordable than urokinase (4,000 INR) and alteplase (49,000 INR). In resource-limited settings, where financial considerations play a pivotal role, attempting catheter clearance with streptokinase becomes a reasonable choice. Even in developed countries like Australia, a study has demonstrated the high cost versus low efficacy rates of alteplase, the standard FDA-approved drug [12].

The dosing method for streptokinase has been evolving over time. Studies by Welik et al. [11] first demonstrated prolonged infusions of 1,000 to 2,000 units per hour for 48 hours, even though efficacious, required prolonged hospitalization, but recent studies by Tahir et al. [13] from Pakistan and Shrestha et al. [14] from Nepal showed promising results with a shorter, higher-dose regimen (25,000 units). Similarly, a study in North India by Paul and Singh [8] used streptokinase at a bolus dose of 50,000 units for occluded central venous catheters (CVCs). In our study, we administered 4,000 units of streptokinase per hour in both ports for eight hours to dilute antigens and minimize febrile reactions.

The study participants had a mean age of 48.2 ± 11.96 years and an average CKD duration of 16.4 ± 13 months, which aligns with demographic characteristics in similar studies from Asia: Tahir et al. [13] from Pakistan (mean age: 44.63 ± 3.782 years, CKD duration: 2.19 ± 6.814 years) and Shrestha et al. [14] from Nepal (mean age: 67.57 ± 12.77 years).

Notably, all participants in our study achieved the primary endpoint of a BFR greater than 300 ml/min (10/10, 100%). This is consistent with findings from previous studies where Welik et al. [11] showed an efficacy of 96.4% and Tahir et al. [13] showed 91% efficacy. Bashardoust et al. reported a similar result in 2017, highlighting the use of streptokinase [15]. While Paul and Singh [8] demonstrated lower efficacy by streptokinase (60%), we believe that the infusion method of drug administration, as opposed to a bolus, could explain the higher efficacy of our regimen.

We followed patients who achieved the primary endpoint for 60 days to assess the patency of the catheter; we found that 80% maintained their patency (8/10), which is similar to the study by Welik et al. [11], who reported that blockade of central catheters led to catheter dysfunction in only 17.6% of cases post-administration of streptokinase infusion. More recently, Paul and Singh [8] used bolus streptokinase in 15 patients with recent onset catheter function, followed for three months, and found patency of only 27%, while the urokinase group in the same study demonstrated 39% efficacy in maintaining patency after three months of follow-up. Paul and Singh [8] selected a more recently blocked CVC (within 24 hours), where causes due to malalignment of the catheter tip were predominant.

The average gain in catheter patency days in our study was consistent with the findings from a study by Shrestha et al. [14], who followed the patency of an occluded perm catheter following streptokinase instillation for six months (patency rates of 80%).

The elderly population is more likely to experience streptokinase adverse effects, particularly bleeding and hypotension [16,17]. Similarly, in our study, we found the incidence of adverse effects to be higher in Group C (mean age: 66 ± 2 years) compared with Group A (mean age: 39 ± 3 years). We suggest exercising precaution in the elderly frail population with streptokinase infusion.

Clase et al. conducted a comprehensive systematic review of catheter thrombolysis, examining studies published up to the year 2000. Their findings suggested that both recombinant tissue plasminogen activator (r-tPA) and urokinase were considered safe and effective in restoring patency to thrombosed hemodialysis catheters. Reports indicated restoration rates ranging from 83% to 98% with the instillation of 1-2 mg/lumen r-tPA [18]. However, studies from Australia and around the world question the cost versus benefits of alteplase as a thrombolytic agent of choice, especially in view of its cost and low efficacy (60%) in blocked tunneled catheters [12].

Our study on the efficacy of low-dose streptokinase infusion for late-onset permanent tunnel catheter dysfunction is significant as it provides valuable insights into a novel therapeutic approach for improving hemodialysis outcomes. By demonstrating the effectiveness and safety of streptokinase in this context, our findings have the potential to influence clinical practice and enhance patient care in resource-limited settings.

The limitations of our study are the relatively small sample size of 10 patients and the single-center study, which may limit the generalizability of the findings in CKD patients. Additionally, the follow-up period of 60 days may not capture long-term outcomes related to catheter patency.

In resource-limited countries, extending the life of a permanent catheter can be lifesaving. Despite its cost-effectiveness, more robust studies are needed, including randomized controlled trials, multicenter trials, and multiethnic trials. Investigating cost-effectiveness and patient outcomes in larger patient populations could provide valuable clinical practice insights.

## Conclusions

Streptokinase is a cost-effective, efficacious, and remarkably safe alternative for late-onset catheter dysfunction in resource-limited settings, especially in young patients. Caution must be exercised in patients with old age and a CKD history of long duration. In third-world countries where tunneled catheters are sometimes the only access, extending their patency can be lifesaving.
